# Analogy, explanation, and proof

**DOI:** 10.3389/fnhum.2014.00867

**Published:** 2014-11-06

**Authors:** John E. Hummel, John Licato, Selmer Bringsjord

**Affiliations:** ^1^Department of Psychology, University of IllinoisUrbana-Champaign, IL, USA; ^2^Department of Computer Science, Rensselaer Polytechnic InstituteTroy, NY, USA; ^3^Department of Cognitive Science, Rensselaer Polytechnic InstituteTroy, NY, USA

**Keywords:** explanation, analogy, logic, modeling, LISA

## Abstract

People are habitual explanation generators. At its most mundane, our propensity to explain allows us to infer that we should not drink milk that smells sour; at the other extreme, it allows us to establish facts (e.g., theorems in mathematical logic) whose truth was not even known prior to the existence of the explanation (proof). What do the cognitive operations underlying the inference that the milk is sour have in common with the proof that, say, the square root of two is irrational? Our ability to generate explanations bears striking similarities to our ability to make analogies. Both reflect a capacity to generate inferences and generalizations that go beyond the featural similarities between a novel problem and familiar problems in terms of which the novel problem may be understood. However, a notable difference between analogy-making and explanation-generation is that the former is a process in which a single *source* situation is used to reason about a single *target*, whereas the latter often requires the reasoner to integrate multiple sources of knowledge. This seemingly small difference poses a challenge to the task of marshaling our understanding of analogical reasoning to understanding explanation. We describe a model of explanation, derived from a model of analogy, adapted to permit systematic violations of this one-to-one mapping constraint. Simulation results demonstrate that the resulting model can generate explanations for novel explananda and that, like the explanations generated by human reasoners, these explanations vary in their coherence.

## Introduction

People constantly seek, generate, and evaluate explanations (Thagard, 1989, 2012; Sloman, 2005; Keil, 2006). At its most mundane, our propensity to explain guides our simplest actions, as when we decide to throw away sour milk: “The milk smells sour because it has gone bad.” At the other extreme, explanation (*aka*, *abduction*; see Josephson and Josephson, 1994; Magnani, 2009) lies at the heart of our most uniquely human endeavors, including science, engineering and mathematics. And in between, it helps us to understand why a street might be closed or why people in Kansas tend to vote Republican. As anyone who has ever given an essay exam knows, the ability to explain is also a powerful index of understanding.

What all these activities have in common is that they are largely inductive exercises^1^ : Our inference that the milk has gone bad is based on our previous experiences with spoiled milk, rather than the fact that sour-smelling milk is logically guaranteed to be spoiled (consider, e.g., buttermilk). Scientific theories are similarly inductive in nature. Observations consistent with a theory add to the evidence that the theory is correct, but they do not logically *prove* it correct (a fact known as the *problem of scientific induction*). Even our species' most purely deductive endeavor, logical theorem proving, has an element of induction at its base: A mathematician's proof of a theorem may consist entirely of deductive reasoning from the premises to the conclusion, but the theorem itself likely arose from less formal, more inductive (perhaps even “intuitive”), origins.

These considerations suggest a common set of inductive mechanisms may underlie all our explanatory behaviors, from throwing away the milk, to discovering and refining the theory of gravity or evolution, to the decision that Poincare's Conjecture or Gödel's Incompleteness Theorems are worth trying to prove.

All animals are capable of inductive inference. Even a rat will freeze at the presentation of a tone that has been paired with a shock, or press a lever in anticipation of a food reward. But the human capacity for inductive inference differs qualitatively from the inductive abilities of other primates in our ability to make inferences that depend, not just on the perceptual features of the objects involved, but on the *relations* between those objects (Penn et al., 2008): The fact that a planet orbits a star does not depend on the perceptual “features” of the planet or star, but on the relations between the bodies' masses and distance. And the fact that a log will make a suitable bridge across a stream depends on the relation between the length of the log and the width of the stream, and on the relation between the strength of the log and the weight of the objects we wish to move across the stream.

### Analogy and explanation: the role of flexible relational knowledge representation

Chief among the manifestations of our ability to reason explicitly about relations is our ability to reason using analogies, schemas, and rules (Gick and Holyoak, 1980, 1983; Gentner, 1983; Holyoak and Thagard, 1995; Hummel and Holyoak, 1997, 2003). Indeed, analogy-making is broadly regarded as a *sine qua non* of relational thought (see Gentner, 1983; Holyoak and Thagard, 1995; Doumas et al., 2008). Accordingly, although a detailed, algorithmic account of explanation remains largely elusive (but see Friedman and Forbus, 2008; Hummel et al., 2008; Hummel and Landy, 2009; Landy and Hummel, 2010, for progress in this direction), accounts of explanation generation, use (Ahn et al., 1987; Vosniadou and Brewer, 1987; VanLehn et al., 1992; Patalano et al., 2006), and evaluation (Keil, 2006; Lombrozo and Carey, 2006) rest on assumptions that are common to explanation-generation and analogy-making (e.g., Falkenhainer, 1990).

This paper presents our early attempts to understand, at a detailed algorithmic level, the cognitive operations that underlie our ability to generate explanations. One of our goals in this work is to understand what our decision to throw away spoiled milk has in common with, say, the insights that led Gödel to prove the incompleteness of mathematics (on certain reasonable assumptions). As the empirical literature on explanation generation is comparatively thin, our starting point is one of first principles: What do we know about how people generate explanations, and how can those facts constrain our modeling?

One thing we know about explanation is that it depends on our ability to flexibly access, combine, and apply existing knowledge (Ahn et al., 1987; Vosniadou and Brewer, 1987). This flexibility is illustrated by an experiment by Patalano et al. (2006). In one condition, Patalano et al. gave subjects a novel explanandum of the form “In the population as a whole, people tend to prefer Pepsi to Coke as often as they prefer Coke to Pepsi. However, ministers tend to prefer Coke over Pepsi,” and asked them to explain this new “fact.” Their research subjects generated many different explanations, but one of the typical ones took the general form: “Ministers tend to be conservative. Perhaps the Coke Corporation supports conservative causes.” This explanation reflects a combination of knowledge about ministers, corporations, and the kinds of factors that can lead a person to prefer one product to another, and reflects tremendous flexibility in the way that knowledge is assessed and combined.

The way we generate explanations suggests at least three kinds of flexibility in the representations and processes underlying those explanations. The first is the kind of *relational* flexibility underlying analogical reasoning. For example, one way to account for the “conservative causes” explanation above is to assume that the subject knows that if a person agrees with the political leanings of a company, then that person will tend to prefer the products of that company. Such a schema needs to be relationally flexible in the sense that it needs to be variabilized so that, in the limit, it can be used to reason about any person, product and company.

Second, explanation requires *semantic* flexibility so that it can exploit partial but imperfect matches between the objects and relations composing an explanandum and those in the relevant schemas or examples in LTM. For example, imagine that our experimental subject did not have a “product preference schema” but did know of a prior case in which her friend preferred to use a particular cell phone company because of their liberal-leaning political activism. The subject could use this prior example as a *source* analog (Holyoak and Thagard, 1989) with which to reason about the situation involving ministers and Coke, but only if their mental representations of the situations allowed them to tolerate the semantic differences between their friend, the cell phone company, and the cell phone service on the one hand, and ministers, the Coca Cola Corporation, and Coke on the other (Hummel and Holyoak, 1997).

These same kinds of flexibility also characterize human reasoning using analogies, schemas, and rules (Holyoak and Thagard, 1989, 1995; Falkenhainer, 1990; Hummel and Holyoak, 1997, 2003).

### Beyond analogy: causal knowledge flexible knowledge integration

Although our capacity for explanation shares a great deal with our capacity for analogical reasoning, two additional properties of explanation seem to distinguish it from general-purpose analogy-making. The first concerns the role of causal relations (and related higher-order relations, such as logical entailment). Although higher-order relations play an important, even crucial, role in analogy (e.g., Gentner, 1983; Markman, 1997), analogy-making is nonetheless seen as a general-purpose inference engine, equally happy to operate over all kinds of relational structures. By contrast, causal relations enjoy a privileged organizing role in explanatory structures: It is almost impossible to answer the question *why?* without invoking *because*. As elaborated shortly, we conjecture that, as embodied in explanations, causal relations are neither as implicit as simple “associative links” (as embodied, say, in the Rescorla and Wagner, 1972, model of associative learning, or even in extant Bayesian models of causal inference) but neither as explicit, and thus working-memory (WM)-demanding, as full-blown variabilized relations (Hummel et al., 2008). Rather, they are explicit structures that organize explanations into meaningful parts and guide explanatory reasoning, but which can be held in WM along with the structures they relate.

A second important difference between analogy and explanation concerns the scope of the knowledge structures brought to bear on the solution of a problem. Analogy is typically construed as a process of reasoning about a novel *target* problem or domain in terms of a familiar *source* or *base* domain (Gentner, 1983; Gick and Holyoak, 1983; Holyoak and Thagard, 1989; Hummel and Holyoak, 1997, 2003), and accordingly, extant models of analogy make inferences from a single source to a single target. Of course, multiple source and target analogs may be used for the purposes of inducing a general schema or rule from multiple examples (Gick and Holyoak, 1980, 1983; Gentner and Medina, 1998; Kuehne et al., 2000; Hummel and Holyoak, 2003) or learning a new relation (Doumas et al., 2008). But within any single reasoning episode, reasoning is conceptualized as being based on the mapping from a single source to a single target, and this convention not arbitrary.

The key bottleneck in analogical reasoning is the process of *mapping* the target onto the source: Finding a set of correspondences between the elements (objects, relations, and propositions) of the target and source that systematically reflects the relational structures of both (e.g., Gentner, 1983; Falkenhainer et al., 1989; Holyoak and Thagard, 1989, 1995; Hummel and Holyoak, 1997). Only once these mappings have been established can structurally consistent inferences about the target be drawn from the source. However, this mapping problem is fundamentally ill-posed without assuming constraints on its solution. One of the most basic constraints on mapping—one that is universally accepted among models of analogical reasoning—is the *1:1 mapping constraint*: Each element in the target may correspond to at most one in the source, and vice versa. Even with this constraint, mapping is hard (NP-hard, to be precise), but at least it's solvable; without it, analogical mapping would be hopelessly underconstrained (e.g., Falkenhainer et al., 1989; Holyoak and Thagard, 1989). Consistent with these computational considerations, Markman (1997) showed that human analogy-making is likewise bound by this 1:1 mapping constraint.

Things are not so tidy in the case of explanation. Generating an explanation often requires integrating information from *multiple* sources in LTM. Returning to our ministers and Coke example, the reasoner may have one set of schemas describing the properties of ministers, another describing the conditions under which one's beliefs might lead to particular product preferences, and yet another describing what it means for one person (e.g., a minister) to agree with another person or entity (e.g., the Coke Corporation). In order to generate the “supports conservative causes” explanation, it is necessary to integrate these diverse sources of knowledge, somehow keeping track of what corresponds to what within and between the explanandum and the various schemas. And in order to integrate information from multiple sources in LTM, it is necessary to violate the 1:1 mapping constraint: The minister in the explanandum will correspond to one object in the *product preference* schema and a different one in the *ministers* schema. As elaborated below, we present a solution to this problem that works by serializing the mapping of the explanandum onto the various knowledge structures in LTM. In contrast to the kind of serialization that goes on in the case of schema-, rule-, or relation-induction from multiple examples, the serialization required for explanation must be performed in the service of making inferences about a single target (the explanandum) during a single reasoning episode.

## A process model of explanation

### Knowledge representation

**Figure 1:**
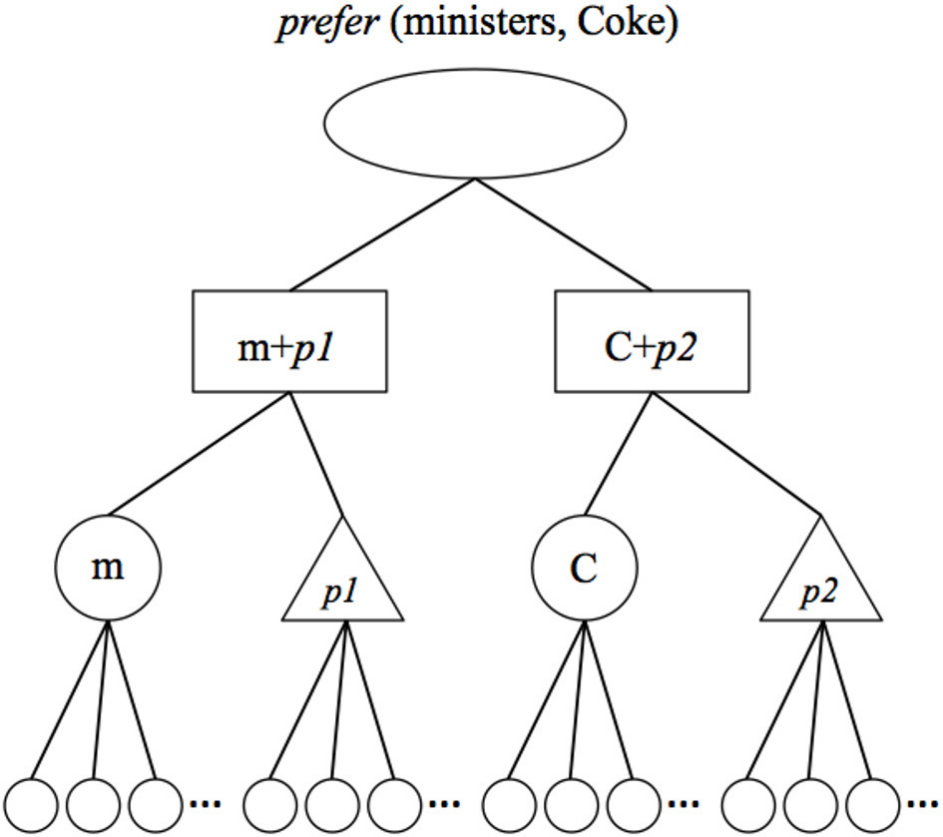
**LISA representation of the proposition *prefer* (ministers, Coke)**. *Semantic* units (small circles) represent the semantic features of objects and relational roles. *Object* and *role* units (large circles and triangles, respectively) represent objects, such as ministers (M) and Coke (C), and relational roles, such as *prefer-agent* (*p1*) and *preferred-thing* (*p2*), in a localist fashion. *Sub-proposition* (SP; aka *role-binding*) units (rectangles) represent bindings of arguments (objects or complete propositions) to relational roles and *proposition* (P) units (oval) represent complete propositions. When a proposition becomes active (i.e., enters working memory), role-filler bindings are represented by synchrony of firing: Separate role bindings (SPs, object, role and their associated semantic units) fire out of synchrony with one another, and units representing the same role binding fire in synchrony with one another.

The point of departure for our effort is Hummel and Holyoak's (1997, 2003) LISA model of analogical reasoning. LISA is an artificial neural network whose representations and processes are rendered symbolic (i.e., explicitly relational) by virtue of its solution to the problem of dynamically binding relational roles to their fillers. LISA represents propositions [such as *prefer* (ministers, Coke)] using a hierarchy of distributed and progressively more localist codes (see Figure 1).

At the bottom of the hierarchy, objects and relational roles are represented as patterns of activation distributed over units coding for their semantic features. At the next level, objects and roles are represented by localist *object* and *role* units, which share bidirectional excitatory connections with the semantic units describing them. For example, the object unit *minister* might share connections with semantics such as *human*, *adult*, *religious*, *official*, etc. Role-filler bindings are encoded by *sub-proposition* units (SPs), which share bi-directional excitatory connections with the object and role units they bind together. At the top of the hierarchy, *proposition* (P) units bind individual role bindings (SPs) together into complete propositions.

This hierarchy represents propositions both in LISA's LTM and, when a proposition becomes active, in its WM. In LTM, a proposition's role bindings are represented strictly by the conjunctive SPs. However, this kind of conjunctive code is inadequate as a general solution to the binding problem in WM, because it fails to represent relational roles independently of their arguments (Hummel and Biederman, 1992; Hummel and Holyoak, 1997; Hummel, 2011). When a proposition becomes active (i.e., enters WM) its role bindings are represented both conjunctively by the SPs, and *dynamically* by synchrony of firing: The separate SPs composing a proposition fire out of synchrony with one another. As a result, relational roles fire in synchrony with the fillers to which they are bound, and separate role-filler bindings fire out of synchrony with one another. On the semantic units, the result is a collection of mutually desynchronized distributed patterns of activation, one for each role-filler binding. These representations have the property that they represent relational roles and their arguments independently of one another (i.e., the same units will represent a given object or relational role, regardless of the role or object to which it happens to be bound at the time) and simultaneously specify how roles are bound to their fillers. They are therefore both distributed and explicitly relational, i.e., symbolic (see Hummel and Holyoak, 1997).

LISA's knowledge representations are compartmentalized into “analogs”: Collections of propositions that together represent complete events, concepts, rules or schemas. Within an analog, a given object or role is represented by a single unit regardless of the number of propositions in which it plays a role. However, separate analogs do not share object, role, SP or P units: A given object or role is represented by one unit in one analog and by a different unit in another analog. As such, object and role units do not represent objects or roles in the abstract; they represent specific instantiations or tokens of those objects or roles in specific analogs. (The same is true of SP and P units.) Accordingly, we will refer to object, role, SP and P units collectively as *token units*. In contrast to the token units, all analogs connect to a common pool of semantic units. The semantic units thus represent the abstract *types* to which the tokens refer.

For the purposes of LISA's operation, analogs are divided into three sets: A *driver* and one or more *recipients* are assumed to reside in *active memory* (a primed subset of LTM that is larger than WM; Cowan, 2001); all others are dormant in LTM. LISA's operations are controlled by the driver. One (or at most three) at a time, propositions in the driver become active and enter the *phase set*: The set of active but mutually de-synchronized role bindings. The phase set is LISA's WM, and like human WM (see Cowan, 2001), is limited to at most 4–6 role bindings at a time. The patterns of activation that propositions in the phase set generate on the semantic units excite other propositions in LISA's LTM (for memory retrieval) and in its active memory (for mapping, analogical inference and schema induction) and thereby bootstrap all the functions LISA performs (see Hummel and Holyoak, 2003, Supplementary Material, for the full details; source code for the 2003 version of the model can be downloaded free from http://internal.psychology.illinois.edu/~jehummel/models.php).

### Processing

Most of the operations performed by the model described here are “standard LISA” and, unless stated otherwise, are performed as described in Hummel and Holyoak (2003) (exceptions to this generalization are described where they become relevant). LISA performs memory retrieval as a form of guided pattern recognition (Hummel and Holyoak, 1997): Patterns of activation generated on the semantic units by one proposition tend to activate other, similar, propositions in LTM, retrieving them into active memory. For example, the patterns activated by the proposition *prefer* (ministers, Coke) might activate the proposition *prefer* (person, product) in the “product preference” schema.

Augmented with a simple algorithm for learning which structures in the recipient tend to activate which structures in the driver, LISA's retrieval algorithm serves as a basis for analogical mapping: In this trivial analogy, *ministers* bound to *prefer-agent* activates *person* bound to *prefer-agent* in the schema, and *Coke* bound to *preferred-object* activates *product* bound to *preferred-object*; as a result, *ministers* fires at the same time as (in synchrony with) *person* and *Coke* fires with *product*, so LISA maps *ministers* to *person* and *Coke* to *product*. The same is true for corresponding roles of the *prefer* relation, and the SP and P units binding those roles to their fillers.

LISA represents these correspondences as learned *mapping connections* between corresponding structures (e.g., between *ministers* and *person*, etc.). These connections serve both to represent the learned mappings and to constrain future mappings. They also play a central role in LISA's capacity for *self-supervised learning*—the core of its algorithm for analogical inference and schema induction (Hummel and Holyoak, 2003).

One of the main adaptive functions of analogical thinking is that it supports *relational generalization*: Inferences based on the relational roles that objects play, rather than just the literal features of the objects themselves. In the current example, once LISA maps *ministers* to *person* and *Coke* to *product* (along with their roles), it is then prepared to “copy with substitution and generation” (Holyoak and Thagard, 1989) the structure of the entire “product preference schema” over onto the “minister and Coke” situation, effectively filling in a (partial) explanation for why ministers might prefer Coke. Through repeated cycles of retrieval, mapping, and inference (elaborated below), the model is able to violate the 1:1 mapping constraint to integrate multiple sources of knowledge through sequential analogical inference.

LISA's knowledge representations (“LISAese”) enjoy the flexibility of distributed representations and the relational sophistication of symbolic representations. As such, they are an ideal platform on which to build a model of understanding and explanation.

### Causal relations

Consider a set of propositions that together might form a “product preference” schema:

P1: *agree-with* (person, corporation)
P2: *produce* (corporation, product)
P3: *prefer* (person, product)

Another set of propositions that might form an “agreement” schema:

P1: *believe* (entity1, proposition)
P2: *believe* (entity2, proposition)
P3: *agree-with* (entity1, entity2)

Assuming that these propositions constitute reasonable caricatures of the preference and agreement schemas, they are clearly causally related to one another. Specifically, P1 and P2 (*agree-with* and *produce*) in the preference schema jointly cause P3 (*prefer*), and P1 and P2 (*believe*) in the agreement schema jointly cause P3 (*agree-with*). How should these causal relations be represented for the purposes of generating explanations?

One straightforward approach is to represent them as explicit propositions, for example:

P4: *and* (P1, P2)
P5: *cause* (P4, P3)

LISAese makes it possible for one proposition to take another as an argument, so this approach to representing causal relations is perfectly plausible; and in some circumstances, people can undoubtedly do so. However, LISAese assumes that explicit propositions are represented in WM and therefore consume finite WM capacity (specifically, when the propositions become active, all their roles must fire out of synchrony with one another, each role occupying a “slot” in WM). As such, we suggest that this approach is likely to be too demanding of WM capacity to serve as a general solution to the problem of representing causal relations for the purposes of explanation: Note that P4 and P5 collectively introduce four additional role bindings into each schema; that's eight additional role bindings that would need to occupy slots (although not all at the same time) in our intrinsically capacity-limited WM. It seems intuitive that, although we are aware of the causal relations, and can name them when asked, we do not necessarily think so explicitly about them in the service of generating an explanation.

Alternatively, we could represent causal relations in an entirely implicit fashion, for example as associative links whose weights indicate causal strength (e.g., as in a Bayes net). This approach would solve the WM problem caused by the explicit propositions, but it goes too far in the opposite direction, representing causal relations only as implicit links rather than explicit structures that can be activated, analogically mapped, and ultimately inferred (e.g., by analogical inference) into the emerging explanation.

**Figure 2:**
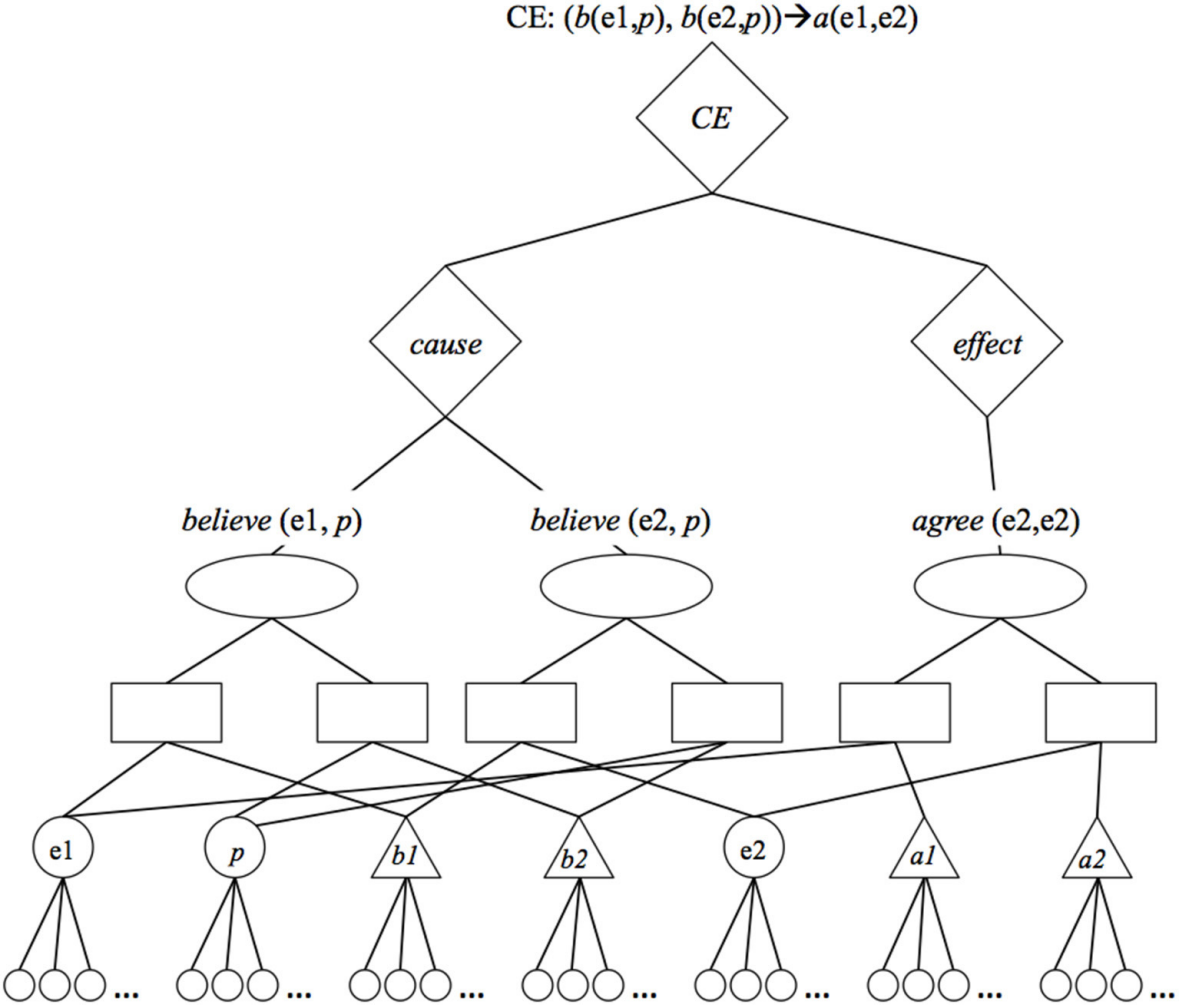
**LISA representation of the cause-effect relation: Entity 1 believes proposition *p [believe (e1, p)]*, entity 2 believes proposition *p [believe (e2, p)]*, and these facts jointly cause e1 to agree with e2 [*agree* (e1, e2)]**. To represent that *believe* (e1, *p*) and *believe* (e2, *p*) jointly cause something, the units representing these propositions (left-most ovals) share bi-directional excitatory connections to a unit (left-most diamond) representing a *cause* group. To represent that *agree* (e1, e2) is the effect of something, the unit representing that proposition (right-most oval) shares a bi-directional excitatory connection to a unit (right-most diamond) representing an *effect* group. To represent that the cause on the left is the cause of the effect on the right, the corresponding cause and effect groups share bi-directional excitatory connections with a unit (upper-most diamond) representing a *cause-effect* (*CE*) group. Connections between the group units and their respective *cause*, *effect*, and *CE* semantic units are not shown.

We propose a third alternative: To represent groups of related propositions by connecting them to *group* units (Hummel et al., 2008). For example, the fact that P1 and P2 in the agreement schema (the *believe* relations) jointly cause something can be represented by connecting P1 and P2 to a single group unit, and tagging that group as a *cause* by connecting it to semantic units representing *cause* (see Figure 2). Likewise, the fact that P3 is an effect can be represented by connecting it to a group unit, and connecting that unit to semantic units representing *effect*. Finally, the fact that the P1/P2 group is the cause of P3 can be represented by connecting the cause and effect groups to a higher-level *cause-effect* (CE) group unit. This latter unit represents the strength of the causal relation by connecting to semantic units coding for that strength.

The resulting representation is more explicit than simply representing causal relations as associative links: causal relations are represented as collections of units that can be activated, mapped and inferred. But at the same time, it is less WM-demanding than explicit propositions: Because group units reside “above” P units in the representational hierarchy (and are effectively different “data types”), they incur no additional WM burden over and above the propositions they link as causally related. (Of course, the notion of units as “data types” is metaphorical. All that matters is that the cognitive architecture respect the units' different spots in the representational hierarchy; see Doumas et al., 2008).

Group units also serve to organize LISA's knowledge into meaningful packages, including but not limited to causes, effects, and cause-effect pairings. As a result, they play a central role in determining which propositions are likely to become active in close temporal proximity (i.e., what LISA is likely to “think about” in what order; see Hummel and Holyoak, 1997, 2003). Specifically, LISA's processing is constrained to activate propositions in group-based sets (in the driver), and it is constrained to retrieve propositions from LTM in group-based sets (a departure from the original LISA; Hummel and Holyoak, 1997, 2003). During memory retrieval, the probability of a group being retrieved into WM at any instant, *t*, is proportional to the group's activation at time *t*. If a cause or effect group gets retrieved, then that event automatically triggers retrieval of the group's parent CE group. As a result, if LISA is reminded of a familiar effect (e.g., some novel explanandum activates a proposition in LTM connected to an effect group), then it will tend to be reminded of the cause as well (via the shared CE group). Thus, group units not only play an important role in LISA's representation of causal relations; they also play a key role in its metacognition, controlling what it “thinks about” together, and controlling what it is reminded of together.

### Flow of control

Armed with group-augmented LISAese, LISA's algorithm for explanation operates according to a retrieve-map-infer cycle that is applied iteratively to construct a causal chain representing an explanation of the explanandum (see Table 1). (The same retrieve-map-infer process also characterizes reasoning by analogy (e.g., Gentner, 1983; Holyoak and Thagard, 1989; Hummel and Holyoak, 2003); but in analogical reasoning, it is performed only once, not iteratively). This process can be initiated by placing the proposition(s) representing the explanandum into the driver, connected to an isolated effect group (i.e., an effect group with no parent CE group and no sibling cause group). All of LISA's other knowledge resides dormant in LTM. In the case of our ministers and Coke example, the driver would contain the proposition *prefer* (ministers, Coke) connected to an effect group; LTM would contain all of LISA's other knowledge, including the preference and agreement schemas. (In general, it is not necessary to connect the propositions of the explanandum to an effect group, as elaborated in the Simulations, but doing so is a convenient way to mark the explanandum as *that which is to be explained*.)

**Table 1:** **Illustration of the retrieve-map-infer cycle that governs explanation-generation in LISA**.

**Cycle**	**Retrieval Cue**	**Retrieved**	**Mappings**	**Inference**
0	*prefer* (ministers, Coke)	*prefer* (person, product)	*prefer1* → *prefer1*	*agree-with* (min., corp.)
		(from *Product Preference Schema*)	*prefer2* → *prefer2*	*prod*uce (corp., Coke)
			ministers → person	
			Coke → product	
1	*agree-with* (min., corp.)	*agree-with* (ent1, ent2)	agree1 → agree1	*believe* (min., prop.)
		(from *Agreement Schema*)	agree2 → agree2	*believe* (corp., prop.)
			ministers → entity1	
			corporation → entity2	

LISA initiates the explanation process by activating the proposition and its effect group in the attempt to retrieve a relevant schema or prior example from LTM. In the case of the current example, the isolated effect group activates the *effect* semantic, and the *prefer* proposition activates the semantics of *ministers+prefer-agent* and *Coke+preferred-object* (the semantics of *ministers* fire in synchrony with those of *prefer-agent* and out of synchrony with those of *Coke* and *preferred-object*, but all these units fire in synchrony with *effect*). The resulting patterns of activation on the semantic units represent the query *Why do ministers prefer Coke?*, and tend to activate effect groups (via the *effect* semantic) connected to semantically similar propositions in LTM (via the semantics connected to the proposition).

In the current example, P3 in the preference schema, *prefer* (person, product), is likely to be retrieved. Since retrieval is group-based, with a bias in favor of retrieving CE groups over isolated cause or effect groups, the activation of P3 in the preference schema is likely to result in the retrieval of the whole product preference schema. (For convenience, we illustrate flow of control in LISA using the preference and agreement schemas, but the logic is exactly the same if, instead of schemas, LISA has analogous specific examples.)

If LISA fails to retrieve a CE group from LTM, then it halts, declaring the explanation complete. (If the resulting explanation is the empty set, then LISA's answer is effectively “I don't know.”) If LISA succeeds in retrieving a CE group, then it places a proxy of that group into a *workspace.* That is, it copies the units composing the group into a new target analog, which we assume to correspond to neurons in frontal cortex with rapidly modifiable synapses (see Knowlton et al., 2012). Retrieval is thus a matter of activation and proxy creation rather than simply activating an existing structure in LTM (another important departure from Hummel and Holyoak, 1997, 2003). LISA then maps the elements of the explanandum onto the proxy of the CE group, for example, mapping *ministers* onto *person*, *Coke* onto *product*, and *prefer* onto *prefer* (along with their SPs, P units and effect groups; see Table 1).

The model next makes the workspace the driver and the explanandum the recipient and, using analogical inference (i.e., self-supervised learning; Hummel and Holyoak, 2003), infers the missing elements in the explanation. In this case, it would infer:

P2: *agree-with* (ministers, corporation)
P3: *produce* (corporation, Coke).

In making these inferences, it will connect both P2 and P3 to a cause group (also inferred from the workspace), and connect both that cause group and the existing effect group [containing P1: *prefer* (ministers, Coke)] to a CE group. LISA's explanation now consists of the hypothesis “ministers prefer Coke because they agree with the corporation that makes Coke.”

Finally, LISA attaches both P2 (*agree-with*) and P3 (*produce*) to their own effect groups, turns control back over to the explanandum (which is now an emerging explanation) and starts the whole cycle over again. Attaching P2 and P3 to effect groups is LISA's way of seeking new causes to explain these facts: *Why do ministers agree with the Coke corporation?* (P2) and (less sensibly) *Why does the Coke corporation produce Coke?* (P3). When the effect group connected to P2: *agree-with* (ministers, corporation) is used to drive retrieval, the result is likely to be retrieval of the agreement schema (or an analogous specific example), in which case the same processes described above augment the explanation with the statements:

P4: *believe* (ministers, some-proposition)
P5: *believe* (corporation, some-proposition),

Connecting both P4 and P5 to a cause group linked via a CE group to the effect group connected to P3 (*agree-with*).

In the current instantiation of the model, these processes are repeated until the retrieval phase fails to retrieve a CE group. This “explanation is done when retrieval fails” approach is a clear limitation of the model in its current state.

What is important to point out in the preceding description of the flow of control is the model's solution to the 1:1 mapping problem: LISA mapped *ministers* to *person* in the context of the preference schema, and then mapped *ministers* to *person* (a completely different token) in the agreement schema. It then inferred *corporation* from the preference schema into the explanandum and then mapped *corporation* onto *entity* in the agreement schema. How did it “know” that *corporation* in the preference schema had the same referent as *entity* in the agreement schema, or that *person* in the preference schema had the same referent as *person* in the agreement schema? The answer is that it did not know, and it did not have to. Rather than having to make the impossible decision of whether two tokens have the same referent, LISA's iterative retrieve-map-infer algorithm need only decide whether two units *correspond*, that is, map to one another, within the confines of the *current* retrieve-map-infer cycle. In so doing, it side-steps the question of whether the tokens “have the same referent.” In short, LISA replaces the question “are they the same?” with the question “do they correspond?” and in so doing provides an effective solution to one particularly thorny variant of the type-token problem. Its ability to do so is a cornerstone of its ability to integrate multiple diverse sources of knowledge in LTM in the service of explaining a novel explanandum.

## Simulations

The model described thus far is still in an early stage of development. In order to test its potential, we ran three sets of simulations. The first two were based on elaborations of the minister/Coke example given previously, and the third was directed as a small first step to understanding how the processes of analogy and explanation might manifest themselves in the domain of mathematical theorem proving—specifically, Gödel's First Incompleteness Theorem (GI).

### Simulation 1: why do ministers prefer coke?

Our first simulations were designed to explore the model's ability to explain why a novel (but nonetheless fairly mundane) explanandum, such as the assertion that ministers prefer Coke to Pepsi. In these simulations, the explanandum was the statement that ministers prefer Coke. We placed several schemas into LISA's LTM (see the Supplementary Material for the simulation details): (1) A (partial) *preference* schema stated that a person may prefer some manufacturer's product because they agree with (e.g., the politics of) that manufacturer. (2) A *minister* schema specified various properties of ministers. And (3) an *agreement* schema specified that if a person supports some (e.g., political) cause and some other entity supports the same cause, then that person and entity agree with one another. We also seeded LISA's LTM with an irrelevant story about a person driving to the beach so that we could evaluate the selectivity of the model's retrieval process.

We ran this simulation several times, and the model produced explanations of varying quality. A typical result was an explanation such as:

P1: *prefer* (ministers, Coke)
P2: *support* (ministers, some-cause)
P3: *support* (corporation, some-cause)
P4: *agree-with* (ministers, corporation)
P5: *manufacture* (corporation, Coke)
*cause* (P2, P3) (P4)
*cause* (P4, P5) (P1)

where P1 is the explanandum and “*cause*” is shorthand for a collection of cause, effect, and CE groups; the first pair of parentheses on each line enclose the P units connected to the cause group and the second pair enclose the propositions connected to the effect group. In other words, LISA inferred that: ministers support some cause (P2); the corporation that makes Coke (P5) supports the same cause (P3); these facts together cause the minister to agree with the corporation [*cause* (P2, P3) (P4)]; and this agreement, along with the fact that the company manufactures Coke, causes the ministers to prefer Coke [*cause* (P4, P5) (P1)].

This explanation represents the most typical result of the pilot runs. The model sometimes also produced a truncated “explanation” in which the ministers are assumed to agree with the Coke corporation, but the model failed to infer why. This explanation obtains when the explanandum, *prefer* (ministers, Coke), retrieves the preference schema on the first retrieve-map-infer cycle, but fails to retrieve anything on the next cycle.

A third result obtained when the explanandum retrieved nothing even on its first retrieval cycle. In this case, LISA halted without generating any explanation at all (effectively saying, “I don't know”).

Finally, the model occasionally retrieved the agreement schema (rather than the preference schema) on the first retrieval attempt. In this case, because analogical mappings are relationally flexible, *ministers* maps to *person*, and *Coke* maps successfully (but nonsensically) to *entity*. In this case, the model generates the nonsensical “explanation”:

P1: *prefer* (ministers, Coke)
P2: *support* (ministers, some-cause)
P3: *support* (Coke, some-cause)

In no cases did the model retrieve completely irrelevant information from LTM (e.g., about Bill driving to the beach), illustrating that the algorithm is capable of selectively retrieving and mapping only situation-relevant information. That said, both its successes and its failures can be traced directly to the success or failure of the retrieval stage: LISA's mappings and inferences on a given retrieve-map-infer cycle will follow structurally from whatever it retrieves during the *retrieve* phase of this cycle. If it retrieves something sensible, then its inferences will be sensible; if not, then its inferences will be less sensible or even nonsensical. It is a sharp limitation of the model in its current state that it cannot evaluate, for itself, which of these is the case.

### Simulation 2: why do ministers dislike coke?

The results of the first simulations were informative for the purposes of exploring the model's properties, but in order to quantify the model's behavior, we ran a suite of 60 additional simulations during which we more carefully tabulated the simulation results. On these simulations, the explanandum was the statement “ministers dislike Coke” (a proxy for “ministers prefer Pepsi”). These simulations replaced the *agreement* schema from the first simulations with a *disagreement* schema and included an additional schema specifying that Coke used to contain cocaine (see the Supplementary Material for details).

Table 2 summarizes the results of Simulation 2. On 16 of the 60 runs, the model produced no explanations (by failing to retrieve anything from LTM on the first cycle), and on an additional 10 runs it made inferences without causally connecting those inferences to the explanandum. In each of the latter 10 cases, the model simply asserted that ministers disagree with some entity, and that that entity supports Coke (the beverage, not the corporation). This response is analogous to LISA declaring, “some people support Coke and ministers disagree with those people.” Inasmuch as this assertion is not an explanation of *why* ministers dislike Coke (or disagree with those people) it is perhaps strangely appropriate that LISA did not embed the constituent propositions inside cause and effect groups. It is also tempting to observe that this general form of “explanation”—simply restating the original question in different terms—is not uncommon in human interactions (e.g., answering “Because it's true!” when asked, “Why do you believe *x*?”). Part of understanding explanation is explaining the kinds of explanations people are likely to offer, including the bad ones.

**Table 2:** **Summary of the number (*n*) of each category of explanation the model generated in Simulation 2**.

**Explanation category**	***n***
**Nothing (“I don't know”)**	**16**
**Assertions without causes**	**10**
• *disagree-with* (minister, entity), *support* (entity, Coke)	
**One causal link**	**8**
• *contained* (Coke, cocaine) → *dislike* (minister, Coke)	(5)
• *immoral* (Coke) →*dislike* (minister, Coke)	(3)
**Two causal links**	**8**
• (*contained* (Coke, cocaine), *illegal* (cocaine))→ * dislike* (minister, Coke)	(5)
(*contained* (Coke, cocaine), *manuf*. (Coke-corp., Coke)) → *contained*(Coke-corp.)	
• *immoral* (Coke)→ *dislike* (minister, Coke)	(1)
*contained* (Coke, cocaine)→ *immoral* (Coke)	
• (*contained* (Coke, cocaine), *illegal* (cocaine))→ *dislike* (minister, Coke)	(2)
*contained* (Coke, cocaine)→ *contained*(Coke-corp.)	
**Three causal links**	**18**
• *immoral* (Coke)→ *dislike* (minister, Coke)	(15)
(*contained* (Coke, cocaine), *illegal* (cocaine)) *immoral* (Coke)	
(*immoral* (Coke), *manufacture* (Coke-corp., Coke)) →*immoral* (Coke-corp.)	
• *immoral* (Coke)→ *dislike* (minister, Coke)	(2)
(*contained* (Coke, cocaine), *illegal* (cocaine)) * immoral* (Coke)	
* immoral* (Coke) → *immoral* (Coke-corp.)	
• (*contained* (Coke, cocaine), *illegal* (cocaine)) →* dislike* (minister, Coke)	(1)
* illegal* (cocaine) →*dislike* (minister, cocaine)	
(*contained* (Coke, cocaine), *manu.* (Coke-corp., Coke))→ *contained* (Coke-corp.)	

Right-facing arrows indicate causal relations. Propositions nested within parentheses act as joint causes.

On eight runs, the model generated short explanations consisting of a single causal link: either “ministers dislike Coke because Coke used to contain cocaine” (5 runs) or “ministers dislike Coke because Coke is immoral” (3 runs). On an additional eight runs, it generated explanations consisting of two causal links, for example, “ministers dislike Coke because Coke is immoral, and Coke is immoral because it used to contain cocaine.” The remaining 18 runs resulted in explanations consisting of thee causal relations. The most common (15 runs) asserted that “Ministers dislike Coke because Coke is immoral. Coke is immoral because it used to contain cocaine. The Coke Corporation is immoral because Coke is immoral and the Coke Corporation manufactures Coke.” This last statement (that the Coke Corporation is immoral) seems unnecessary to the logic of the explanation, and demonstrates that the algorithm is able to pursue causal chains that do not strictly lead to the explanandum. Interpreted colloquially, this behavior resembles the model adding a parenthetical aside (“Oh, by the way, this also implies that the Coke Corporation is immoral.”).

### Proof generation as explanation

In logic and mathematics, and the formal sciences generally, exquisite, definitive explanations are routinely provided via proofs. (This empirical fact leaves perfectly intact the important observation that in many disciplines, deduction doesn't explain; and indeed leaves intact the specific empirical fact that much deduction has no explanatory value in many contexts, scientifically speaking: A disjunction of *P* or *Q* follows deductively from *P*, and this theorem is crucial in proofs of *P* or *not-P*, but the theorem is manifestly empty in many contexts.) The Incompleteness Theorems are themselves a case in point, for until one sees how the proofs work, one cannot really understand in what senses these results are limatative, and until one understands these senses, one cannot understand what the theorems in question, in broad context, tell us.

The mechanisms used for analogy and explanation generation can also be implicated in the generation of logico-mathematical proofs. One might think of a proof as a type of explanation where instead of having causally-connected explanations, the individual progression of beliefs is connected by accepted inference rules in some logical system. (Because causal and logical relations have similar syntactic forms, and because LISA's cause, effect and CE groups are equally suited to represent both, we will, for convenience, simply refer to such relations as “causal,” bearing in mind that causal and logical relations have important semantic differences.) The generation of such proofs, then, can be aided by analogy in at least two ways: First, the mechanism described earlier for retrieving source analogs might be used in retrieving a relevant logical inference rule. Alternately, analogy may be used to transfer high-level *strategies* from one domain to another.

As an example, consider the highly influential Incompleteness Theorems of Kurt Gödel. The First Incompleteness Theorem, which we will refer to as G1, places fundamental limitations on any finitely formal theory that is expressive enough to capture ordinary arithmetic.^2^ How could Gödel have devised such a brilliant proof in the first place?

Of course, we can never know precisely what it was that allowed Gödel to figure out what he did, except to note that Gödel mustered plenty of extra-analogical innovation to accomplish his Incompleteness Theorems. At most, we can speculate based on the mathematical knowledge that was already well-known to Gödel at the time [see Ebbinghaus et al. (1994), which is regarded to be a description of G1 that is quite close to Gödel's original work], but as we are not interested here in the details of the intellectual history of mathematical logic, we will be very brief. One plausible speculation is that the initial insight that led to G1's conjecturing came from an analogy to a simpler problem. Gödel was described as having a thought that moved “from conjecture to conjecture,” even when he was not quite sure “how (or whether it is possible) to bridge the gap between them” (Wang, 1995, p. 184). In fact, there is a suitable source analog, known as the Liar Paradox (LP). The simplest form of LP consists of a single proposition *l*: “This sentence is a lie.” One runs into trouble when attempting to determine the truth value of *l:* If it is true, then it is a lie, and therefore false; but if it is false, then since it says it is false, it's true. What are we to do with a sentence that seemingly is neither true nor false, but is still somehow meaningful?

A careful analysis of *l* will allow us to make some useful inferences. Since *l* is self-referring, we might take this to constitute a simple existence proof of a property of the English language: that it is self-referable, meaning that it allows for the formulation of a self-referring statement such as *l*. Our failed attempt to assign a truth value to *l* also tells us that English has at least one unverifiable statement. The fact that such a statement exists means, by definition, that English is *logically incomplete*. Let us name the three properties we just deduced of English as: *self-referable*, *has-unprovable-statement*, and *incomplete*, respectively.

The analogy that allows a high-level proof-sketch to transfer from LP to G1 can then be sketched as follows. We have a source analog consisting of the knowledge that our analysis of *l* provided:

P1: *self-referable* (English)
P2: has-unprovable-statement (English)
P3: *incomplete* (English)
*cause* (P1) (P2)
*cause* (P2) (P3)

We can then fill out a very minimal target analog, consisting of only a single proposition:

Q3: incomplete (Σ)

Here Σ represents any theory of interest; in this case Σ is ultimately going to range over all formal theories of arithmetic that have certain basic properties. That, however, is not relevant for this particular analogical match. Rather, all we are asking LISA to do is tell us: If we want to show that some theory Σ is incomplete, what do we have to do?

The resulting inference retrieves concepts from the domain to which Σ belongs, which in our hypothetical example is the formal logico-mathematical domain. A successful analogical match, then, will match the predicates used in our source analog to the very rigorously defined formal concepts in the target domain. Finally, the resulting set of analogical inferences give a high-level description of how to prove Q1.

When we ran LISA with the source (P1… P3, along with their causal relations) and explanandum (Q3) described above, it generated the explanation:

Q1: self-referable (Σ)
Q2: has-unprovable-statement (Σ)
Q3: incomplete (Σ)
*cause* (Q1) (Q2)
*cause* (Q2) (Q3)

It would also be plausible to assume that instead of starting with Q3 in the target, we start with nothing but Q1. We ran this simulation as well, and LISA again generated the correct explanation. Essentially, these analogical inferences would have told Gödel that in order to show incompleteness for Σ, he would have to show that Σ has an unprovable sentence, which he would in turn be able to show by exploring whether Σ can be self-referring.^3^.

What we have just described is an overview of what has been called Analogico-Deductive Reasoning (ADR), or the combination of analogical and hypothetico-deductive reasoning (Bringsjord and Licato, 2012; Licato et al., 2012). In ADR, an analogical inference is used to generate some hypothesis in the target domain, and subsequent deductive reasoning is used to either deductively prove this hypothesis, refute it by deriving a contradiction, or suggest an experiment and an expected outcome of the experiment which would then either support or refute the hypothesis. ADR is just another example of how analogy can be used to generate an understanding of the world, whether through explanation, or through formal proofs, as in the LP-to-G1 example.

For a more detailed example modeling the analogy from LP to G1, see Licato et al. (2013). We conclude this example by noting that although analogy may have been useful in pointing Gödel toward the insight that LP may be useful in proving G1, *much* more work was necessary before his proofs could be considered complete, e.g., the creation of Gödel numbering, the formalization of effective procedures, etc.

## Discussion

We described our progress toward a process model of explanation. The model is based on a model of analogy (Hummel and Holyoak's, 1997, 2003, LISA), reflecting our assumption that many of the core processes of explanation are also core processes of analogy making.

However, modeling explanation necessitates going beyond modeling analogy in at least two important respects: First, explanation, much more than analogy, depends on causal relations. We model the representation of causal relations using units representing groups of propositions (and other groups). This representational format is more explicit than simple associative links between causes and effects (as in Bayesian models, e.g., Tenenbaum et al., 2006), but less explicit than propositions about cause and effect. It permits the model to use cause, effect, and cause-effect groups as units of both cognitive control and memory retrieval.

Second, explanation, unlike analogy, often requires the reasoner to integrate information from diverse sources in LTM, which in turn requires the reasoner to violate the 1:1 mapping constraint. We resolve this difficulty by serializing the process of incorporating facts from different sources in LTM.

Preliminary simulation results suggest that the approach is a promising approach to modeling explanation, and indeed, to the problem of understanding more broadly.

That said, the model is in an early stage of development, and many problems remain to be solved before we have a complete (much less correct) process model of explanation. First, we must address the problem of how a human reasoner knows when an explanation is complete. In the current model, this decision is based strictly on the failure to retrieve additional causes from LTM. This is clearly incomplete, but what is right is harder to say. Second, we must address the problem of explanation evaluation (for progress in this direction, see Thagard, 2001). One of the hard problems to be solved in this domain is contradiction detection: How does the cognitive architecture know when it has postulated something just plain stupid [e.g., “*believe* (Coke, some-proposition)”]? Third, we must include a role for elaboration in explanation: in our example problem, for instance, the model is given the knowledge that ministers are politically conservative, but the model never suggests that the source of agreement between the Coke corporation and the ministers is one of conservative values. Such elaboration is not part of the causal chain approach here, but seems to be a central component of explanation generation.

These issues remain serious hurdles in our attempt to understand how people generate explanations. In the meantime, we believe our current work takes us a step closer to understanding the basic processes underlying explanation, from inferences/hypotheses as simple as why ministers might prefer Coke, to conjectures about theorems that might be possible and worthwhile to prove.

### Conflict of interest statement

The Associate Editor Aron K. Barbey declares that, despite being affiliated to the same institution as author John Hummel, the review process was handled objectively and no conflict of interest exists. The authors declare that the research was conducted in the absence of any commercial or financial relationships that could be construed as a potential conflict of interest.

## Footnotes

^1^In practice, it is difficult to distinguish inductive inferences from deductive inferences from faulty axioms. If you misidentify a car in the parking lot as “your car,” is this an inductive inference (which is not guaranteed to be right, even given correct premises and a syntactically valid inferential form) or a (valid) deductive inference from faulty premises? In this paper, we shall refer to inferences of this kind, and of the kind underlying analogy and explanation in general, as *inductive* to emphasize the fact that they are not guaranteed to be right and the fact that they are typically based more on specific experiences than a-priori axioms.

^2^We leave out a more precise description of Gödel's Incompleteness Theorems because they are not directly relevant to the purposes of this paper.

^3^Note that self-reference is not the only way to show incompleteness, or even the only way to get a version of the Liar Paradox. For example, suppose one has the two sentences: The sentence below is false. The sentence above is true. These two sentences form a paradox that is similar to LP, without exploiting (direct) self-reference. Here, however, we are only concerned with one route that Gödel might well have used to discover incompleteness
