# Supplementary material for: Analogy, explanation, and proof
Source: Front Hum Neurosci. 2014 Nov 6;8:867. doi: 10.3389/fnhum.2014.00867 (PMC4222223; doi:10.3389/fnhum.2014.00867)
Supplement: Supplementary file 1 [file Presentation1.PDF]

Appendix: Simulation Details

This appendix details the representations used in all the simulations. All simulations were run as described in Hummel and Holyoak (2003), except as detailed here and in the main text. Simulations are described first in terms of the semantic coding of the objects and relations used, then in terms of the propositions and causal relations forming the analogs/schemas in LISA's LTM. In every case, the explanandum was an analog consisting of a single proposition connected to a lone *effect* group (as described in the text). Objects are described on separate lines, with the name of the object unit first (in boldface) followed by a colon and the names of the semantic units to which it was connected. Relations are described in an analogous manner, but with the names of semantics connected to separate roles denoted inside separate pairs of square brackets. To simplify the notation when possible, relation names followed a number and a single list of semantic names (e.g., "**loves**: 2 emotion positive…") indicate the relation has that number of roles and that each role unit is connected to semantic units with the listed names, where each name has the role number appended to its end (e.g., "**loves**: 2 emotion positive…" indicates that the *loves* relation is represented by two role units, **loves1** and **loves2**, where **loves1** is connected to emotion1, positive1, … and **loves2** is connected to emotion2, positive2…). When the name of a semantic unit is followed by '=' with a number (e.g., '=0.1'), the number indicates the value on the weight from the semantic unit to the object or relation unit. If no number is indicated, then the weight was 1.0. The names on the semantic, object and role units are for the benefit of the reader only, and have no meaning to LISA or bearing on its performance, except that units with separate names are in fact separate units inside the model. (That is, names listed below uniquely identify actual units inside the model, so that, for example, if two objects units both list semantic units having identical names, then both objects are connected to the corresponding semantic unit.)

Every simulation was run by placing the explanandum into the driver and allowing it to run, initiating the retrieve, map infer cycle that forms the core of the model's behavior. Aside from the initial retrieve phase (which was initiated by the simulation), each following map and infer phase, as well as subsequent retrieve, map infer cycles, were initiated by the model itself. Each simulation halted automatically when a retrieve phase failed to retrieve any new information from the model's LTM.

Simulation 1: Ministers Prefer Coke

Objects

Bill: human adult male bill1 bill2 bill3
Jeep: artifact vehicle car american jeep1 jeep
beach: location beach commerce recreation ocean sand
Coke: inanimate =0.1 manufactured =0.1 product =0.1 beverage sweet coke
cocaine: inanimate =0.1 manufactured =0.1 drug illegal cocaine opiate addictive
product: inanimate object =0.1 manufactured product
manufacturer: company organization produce make manufacturer
Coke-corp: manufacturer company make coke coke-corp
person: animate object =0.1 human person
entity: organization object =0.1 person

a-cause: abstract goal desire work-toward a-cause
minister: animate =0.1 human =0.1 person minister religious moral
immoral-stuff: object =0.1 ethics morals evil -moral -good immoral immoral-stuff

Relations

has: 2 state has1 has2 has3
goto: 2 ptrans goto1 goto2 goto3
want: 2 state want1 want2 want3
driveto: 3 ptrans goto drive vehicle drive2 drive3
contained: [time=past container holder product] [time=past containee held ingredient]
illegal: [law illegal not-legal immoral]
was-immoral: [time=past ethics evil negative immoral -moral]
manufacture: 2 activity =0.1 make produce manufacture
prefer: 2 mental =0.1 state =0.1 like likemore prefer
support: 2 activity =0.1 help facilitate support
oppose: 2 activity =0.1 help =-1.0 facilitate =-1.0 support =-1.0 dislike oppose
agree-with: 2 mental =0.1 state =0.1 agree
disagree-with: 2 mental =0.1 state =0.1 agree=-1.0 like =-1.0 dislike disagree
dislike: 2 state =0.1 disposition like=-1.0 prefer=-1.0 dislike
like: 2 state =0.1 disposition like dislike=-1.0 prefer
conservative: [state =0.1 political conservative old-school liberal=-1.0 modern=-1.0]
uptight: [state =0.1 old-school -relaxed uptight]
religious: [state =0.1 religion ethics morals religious ignorant willful old-school]
immoral: [property =0.1 ethics morals evil negative moral=-1.0 good=-1.0 immoral]

Analog: Preference Schema

P1: *prefer* (person, product)
P2: *agree-with* (person, manufacturer)
P3: *manufacture* (manufacturer, product)
Cause (P2, P3) (P1) (read this statement as "P2 and P3 jointly cause P1")

Analog: Minister Schema

P1: *uptight* (minister)
P2: *conservative* (minister)
P3: *religious* (minister)
P4: *immoral* (immoral-stuff)
P5: *dislike* (minister, immoral-stuff)
Cause (P4) (P5)

Analog: Agreement Schema

P1: *support* (person, a-cause)
P2: *support* (entity, a-cause)
P3: *agree-with* (person, entity)
Cause (P1, P2) (P3)

Analog: Bill and the beach (an irrelevant analog stored in LTM as a foil)

P1: *has* (Bill, Jeep)
P2: *goto* (Bill, Beach)
P3: *want* (Bill, P2)
P4: *driveto* (Bill, Jeep, Beach)
Cause (P3) (P4)

Analog: Explanandum
P1: *prefer* (minister, Coke)
Effect (P1) (read this statement as "P1 is the effect of something unknown")

Simulation 2: Ministers prefer Pepsi

Simulation 2 was very similar to Simulation 1, except that we replaced the agreement schema of Simulation 1 with a disagreement schema and added a schema specifying that Coke used to contain cocaine. The explanadum specified that ministers dislike Coke.

Analog: Disagreement Schema
P1: *oppose* (person, a-cause)
P2: *support* (entity, a-cause)
P3: *disagree-with* (person, entity)
Cause (P1, P2) (P3)

Analog: Coke and Cocaine
P1: *contained* (Coke, cocaine)
P2: *illegal* (cocaine)
P3: *was-immoral* (Coke)
P4: *manufacture* (Coke-corp, Coke)
P5: *was-immoral* (Coke-corp)
Cause (P1, P2) (P3)
Cause (P3, P4) (P5)

Analog: Explanandum
P1: *dislike* (minister, coke)
Effect (P1)

Simulation3: Incompleteness of second-order logic

Objects
NL: is-theory is-language NL
SOL: is-theory is-language SOL

Relations
allows-self-reference: [sr1 sr2 sr3]
has-unprovable-statement: [hus1 hus2 hus3]

incomplete: [inc1 inc2 inc3]

Analog: Natural language
P1: *allows-self-reference* (NL)
P2: *has-unprovable-statement* (NL)
P3: *incomplete* (NL)
Cause (P1) (P2)
Cause (P2) (P3)

Analog: Second-order logic
P1: *incomplete* (SOL)
